# Insular Pharmacopoeias: Ethnobotanical Characteristics of Medicinal Plants Used on the Adriatic Islands

**DOI:** 10.3389/fphar.2021.623070

**Published:** 2021-05-07

**Authors:** Łukasz Łuczaj, Marija Jug-Dujaković, Katija Dolina, Mirjana Jeričević, Ivana Vitasović-Kosić

**Affiliations:** ^1^ Institute of Biology and Biotechnology, University of Rzeszów, Rzeszów, Poland; ^2^ Institute for Adriatic Crops and Karst Reclamation, Split, Croatia; ^3^ Institute for Marine and Coastal Research, University of Dubrovnik, Dubrovnik, Croatia; ^4^ Independent Researcher, Korčula, Croatia; ^5^ Department of Agricultural Botany, Faculty of Agriculture, University of Zagreb, Zagreb, Croatia

**Keywords:** ethnobotany, traditional medicine, herbal medicine, island biogeography, ethnomedicine, herbal teas, Croatia

## Abstract

The Adriatic islands in Croatia, usually divided into two archipelagos – the Kvarner and Dalmatian islands – is one of the largest groups of islands in Europe. Over 40 islands are still inhabited. Unfortunately the traditional use of medicinal plants was never properly documented there. Our data comes from 343 interviews carried out in 36 islands, including the 15 largest islands of the archipelago. The medicinal plants are mainly used to make herbal infusions or decoctions, occasionally medicinal liqueurs, syrups, compresses, or juices squeezed out of raw plants. We recorded the use of 146 taxa, among them 131 with at least one medicinal purpose and 15 only for tea. The frequency curve of use is relatively steep – several plants are used very frequently and most are reported only by one or two informants, which can be explained both by the large geographical spread of the area, and even more so by the devolution of local knowledge and disappearance of gathering practices due to specialization in tourism, modernization and depopulation. Most of the gathered plants already occur in ancient and medieval herbals and are a part of the pan-Mediterranean pharmacopoeia.

## Introduction

The view that contemporary folk knowledge about medicinal plants in Europe stems mainly from older herbals and contains little indigenous ancient knowledge was first expressed by Józef Rostafiński at the end of the 19th century (Köhler, 2015). Similar reductionist statements have appeared recently (Leonti, 2011). They are however counterbalanced by those who try to document the ethnomedical knowledge for reasons other than only finding new medicinal species. For example, the use of medicinal plants is entwined with issues such as the protection of rare plants and their overharvesting (Barata et al., 2016; Grigoriadou et al., 2020; Papageorgiou et al., 2020). The cultural aspects of healing with the use of herbs are also important and have been summarized by Quave et al. (2012). Plants are not a mere collection of phytochemicals but are set in traditional practices. They have stories and magical meanings attached to them, which can make it difficult to draw the line between ritual and medicinal use (Moerman, 1979; Heinrich, 2000). For example, in Poland, plants are usually blessed on certain occasions and later used for fumigation both in cases of obvious illness easily classified by biomedical science, folk illness (like “fright”), or sheerly to deter bad luck or “evil” forces (Łuczaj, 2012). Nowadays medicinal or herbal tea plants are often collected to “connect to nature” (Grasser et al., 2012). Another broadly discussed issue is the continuum of food and medicine (e.g., Pieroni and Price, 2006; Etkin, 2008). Investigating local uses of plants is also important in the light of growing medical pluralism in Europe (Uibu, 2020).

The study of locally used medicinal plants in the Balkans started with a paper by Leopold Glück, a pharmacist from Sarajevo (Glück, 1892). In recent years a lot of effort was put into the documentation of ethnomedicinal practices in the mainland Balkan Peninsula, especially in remote mountainous areas (Pieroni and Quave, 2014). Such research was carried out in all Balkan countries, including Slovenia (Lumpert and Kreft, 2017; Povšnar et al., 2017; Mattalia et al., 2020); Bosnia-Herzegovina (Šarić-Kundalić et al., 2009; Šarić-Kundalić et al., 2010; Šarić-Kundalić et al., 2011; Ferrier et al., 2015), Serbia (Pieroni et al., 2011; Šavikin et al., 2013; Zlatković et al., 2014; Jarić et al., 2015; Živković et al., 2020), Kosovo (Mustafa et al., 2012; Mustafa et al., 2020); Northern Macedonia (Pieroni et al., 2013; Rexhepi et al., 2013), Greece (Tsioutsiou et al., 2019), Montenegro (Menković et al., 2014), Albania (Pieroni, 2008; Pieroni et al., 2015) and Bulgaria (Ivancheva and Stantcheva, 2000; Nedelcheva and Dogan, 2015). Field research concerning the traditional use of medicinal plants was also carried out in mainland Croatia (Pieroni et al., 2003; Pieroni and Giusti, 2008; Vitasović-Kosić et al., 2017; Varga et al., 2019; Žuna Pfeiffer et al., 2020) but was lacking in the islands. In general, such research in the islands of the eastern part of the Mediterranean Sea has been somewhat neglected. Only Axiotis et al. (2018) studied medicinal plants used in the eastern part of the Egean archipelago of Greece (and found 108 species of plants used there medicinally), Papageorgiou et al. (2020) studied Lemnos, and Bulut (2016) – Marmara Island off the coast of Istanbul.

Previous research on the documentation of traditional use of medicinal plants on Croatian islands is only restricted to a few small ethnographic notes, either listing a few names of medicinal plants on Krk (Žic, 1900) and Korčula (Baničević, 2000), or, in the case of Šolta, mixing local names and local uses with uses from handbooks (Sule, 2010). Several published (printed) herbals and herbal manuscripts have been produced on the territory of present-day Croatia since 1603. For a list and overview, *see* Krnjak (2019) and Kujundžić (2020). However, none of these sources were written on the islands.

Islands have been of great importance in developing ecological and evolutionary concepts but are little studied by ethnobotanists (Łuczaj et al., 2019a; Łuczaj et al., 2019b). The main model explaining biological diversity on islands is the so-called island biogeography theory formulated by MacArthur and Wilson (1967). It has only been tested once in our previous publication on the use of wild vegetables in the same study area (Łuczaj et al., 2019a). The results of this study suggested that the model has little application to ethnobotany of wild vegetables due to greater complexity and fuzziness of knowledge about plants compared to merely biogeographical species distributions of plants or animals. However, we think that it should be tested on more datasets to draw more general conclusions.

One may also wonder to what extend islands tend to preserve more knowledge than the mainland due to a certain degree of population isolation or, quite the contrary, to what extent their pharmacopoeias are degraded due to the effects of island biogeography (such as local knowledge or useful species endangered by dying out on small islands). We may also question whether the islands of the Mediterranean have been isolated at all. In the past, sea transport was very important and often more efficient than land routes, which does not change the fact that Croatian island human populations show an extremely high level of inbreeding (Łuczaj et al., 2019a). In our ethnobotanical research concerning wild vegetables in the Adriatic islands we observed only very weak island biogeography effects and a general geographical trend of decreasing numbers of the species used from Kvarner in the west to eastern Dalmatian islands (Łuczaj et al., 2019a). In the case of plants used to flavor alcoholic drinks, this northwest to southeast pattern was not visible (Łuczaj et al., 2019b). In both studies we found some locally used species with their traditions of use restricted to one or two islands.

In view of the aforementioned phenomena, the aim of our study was to:

1. Record which taxa are used in the local pharmacopoeias in the islands.
2. Establish if such variables as island size, population size, flora or its isolation are correlated with the number of species used.

We formulated a hypothesis that the length of the total medicinal plant list per island, as well as the mean number of species per informant is positively correlated with:

1. The number of species reported in the floras of particular islands. The link between the flora and plant use is obvious: the more species available the more likely it is that more species are used.
2. The area of the island. A larger area within which interviews were carried out meant a larger chance for different species to be found as well as a smaller similarity in village traditions due to larger physical distance between villages.
3. The number of inhabitants. The more people live on the island, the more exchange of knowledge is likely to happen and there are more knowledge holders.
4. The proximity of mainland (i.e., is negatively correlated with the distance from the mainland of Croatia). We assumed that in less isolated islands, whose inhabitants have more social contacts with the mainland, there is more opportunity for the exchange of knowledge.

Hypotheses no. two and four directly test the island biogeography theory and no. one and three result from it indirectly.

## Materials and Methods

**FIGURE 1:**
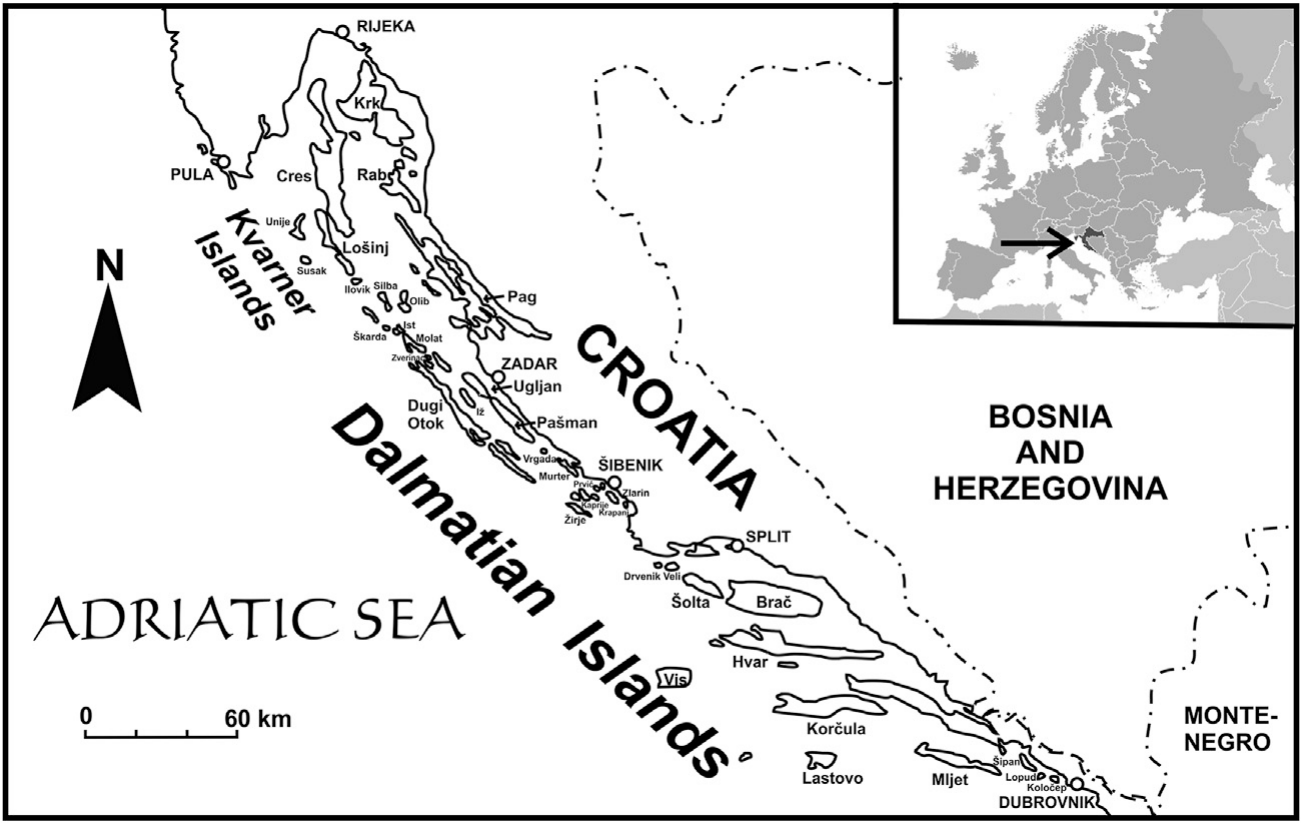
The studied islands. All the named islands are those where interviews were carried out.

The study is part of a larger project on the ethnobotany of the Adriatic islands, from which only data about wild vegetables and plants used to flavor alcoholic drinks have been published so far (Łuczaj et al., 2019a; Łuczaj et al., 2019b). The research was performed between 2013 and 2018, with most interviews carried out in 2016 and 2017. We asked several questions about plant uses (wild edibles, tools, etc.). One of the questions behind these studies concerned the identification of plants that were used medicinally by the respondents or by their relatives in the past. The data in the spreadsheet come from 343 interviews with single people, couples, or rarely groups of a few neighbors. The mean age of respondents was 69 (median 70, minimum 24, maximum 96, 62% were female, 38% male). There are 47 inhabited islands in Croatia. However, some of them are only inhabited in summer, by one person or family, or by newcomers. We managed to interview people from 36 islands, including the fifteen largest islands, each with an area above 40 square km (Figure 1 and Table 1).

**TABLE 1:** List of the studied islands.

Archipelagos and islands	Number of interviews	Total number of species mentioned	Mean number of species listed	Area	Population	Flora (no of species)	Longitude (⁰E)	Isolation (km)
KVARNER ARCHIPELAGO	82	73	4.3					
Cres	17	28	4.1	406	3,079	1,250	14.4	5
Ilovik	8	20	4.9	6	85	n.d	14.6	35
Krk	19	19	2.2	405	19,383	1,170	14.6	0.8
Lošinj	15	45	6.7	74	7,587	1,300	14.4	29
Rab	13	23	4.1	86	9,328	800	14.8	2
Susak	6	11	2.7	4	151	400	14.3	41
Unije	5	26	7.6	17	85	629	14.2	25
ZADAR ARCH.	95	69	5					
Dugi Otok	14	30	4.2	113	1,655	540	15.0	16
Ist	5	7	3.2	20	182	n.d	14.8	26
Iž	5	16	5.4	18	615	n.d	15.1	11
Molat	15	31	5.8	23	197	308	14.9	18
Olib	4	9	5	26	140	465	14.8	26
Pag	15	26	4.9	284	9,059	650	15.4	0.4
Pašman	15	28	4.7	60	2,845	629	15.3	2
Silba	4	18	6.5	15	292	516	14.7	33
Skarda	1	1	1	4	0	n.d	14.7	30
Ugljan	11	21	4.4	51	6,049	n.d	15.2	4
Vrgada	5	25	8	4	249	264	15.5	3
Zverinac	1	5	5	4	43	n.d	14.9	17
ŠIBENIK ARCH.	32	50	5.5					
Kaprije	5	14	4.4	7	189	278	15.7	5
Krapanj	5	14	4.4	0.4	170	257	15.9	0.4
Murter	5	8	2.2	20	3,600	664	15.6	0.1
Prvić	6	31	8.8	2	403	267	15.8	0.8
Zlarin	5	21	6.4	15	103	542	15.7	12
Žirje	6	25	6.2	8	284	444	15.8	1.3
SPLIT ARCH.	64	59	4.9					
Brač	15	26	4.9	395	13,956	750	16.7	5
Drvenik Veli	4	9	3	12	150	n.d	16.4	2
Hvar	17	25	4.5	297	11,077	1,046	16.8	4
Šolta	15	37	5.7	58	1700	267	16.3	15
Vis	12	29	5	90	3,445	598	16.1	43
DUBROVNIK ARCH.	71	88	7.1					
Koločep (Kalamota)	5	13	3.8	2	163	446	18.0	1.5
Korčula	28	66	8.4	271	15,522	858	16.9	1
Lastovo	14	35	5.9	41	792	678	16.9	26
Lopud	4	17	5.7	5	249	429	17.9	3
Mljet	16	41	6.2	98	1,088	712	17.5	8
Šipan	4	20	8.2	16	419	570	17.9	2

The study follows the guidelines of best practices in ethnopharmacological research outlined by Heinrich et al. (2018). We applied the typical methods of ethnobotany: in-depth semi-structured interviews starting from freelisting, and supplemented, if possible, by walks around the places where the respondents gathered plants and could identify the supplied names. Most informants were recommended locally as “those who name/gather plants”. Some informants were encountered directly in the fields or gardens. The interviews were performed in Croatian, the native language of the inhabitants. All the interviewees declare themselves as Croats and speak this language as their first language, apart from a few interviewees from Cres, who use Italian and Croat interchangeably. All the respondents were either Roman-Catholics or at least originated from Roman-Catholic families. The interviews concerned different aspects of plant use, but here we present data only on medicinal and herbal tea uses from respondents who possessed such knowledge. The informants were selected from people who were born on the islands and had their ancestry there. We decided to combine the answers about medicinal plants and recreational teas, as there is a large overlap between them – self-gathered local tea mixes are often drunk for digestion, and the border between tea plants and medicinal plants is difficult to draw (Sõukand and Kalle, 2013). We only included plants directly used by the informants or used by their ancestors in the past. The aim of study (documentation of traditional plant uses) was explained to the respondents. Prior informed oral consent was always sought.

Philips and Gentry’s (1993) use value index (UV) was applied to estimate the cultural importance of species:

$$\mathrm{UV=}\sum U_{i}/N$$

where ${\text{U}}_{\text{i}}$ is the number of different uses mentioned by each informant and N is the total number of informants interviewed in the survey.

In order to look for the sources of variability in the richness of the local pharmacopoeias we correlated the number of species used per single informant and the number of species in the island’s pharmacopoeia with geographical variables such as population size (from 2011), island area (km^2^), shortest distance to the mainland, geographical longitude and the number of species in the flora. The number of species in the flora was extracted from Nikolić et al. (2008) and supplemented by the works of Milović et al. (2016) for Olib and Pandža et al. (2011) for Vrgada.

Statistical analysis was performed using open access PAST software (Hammer et al., 2001). The significance and strength of the relationships between variables was assessed using the Pearson correlation coefficient. Normality of the distribution of variables was tested with the Shapiro-Wilk test. All the variables had normal distribution.

To visualize the similarity in medicinal species lists between the islands, we performed Detrended Component Analysis (DCA) on species level (Hill and Gauch, 1979). We plotted the results of DCA on the two main axes that caused the distribution of the data to visualize potential overlap and variation in the species composition used in different islands. Another way of visualizing the diversity of species composition on different islands was a numeral taxonomy dendrogram obtained by clustering. We used the most common method of clustering, i.e., Unweighted Pair Group Method with Arithmetic Mean (UPGMA), using Euclidean distance (Sokal and Michener, 1958; Bailey, 1994).

Plants were identified using standard floras available in this area of Europe, including guides for the identification of Croatian flora (Domac, 1994; Nikolić, 2019), Pignatti’s Flora of Italy (Pignatti, 1982) and the (Flora Croatica Database). Plant names were updated to be consistent with (The Plant List). Voucher specimens were collected on the islands where they were used, usually with the assistance of the respondents. They were deposited in the herbarium of Warsaw University (WA) with a small subset in the herbarium of the University of Zagreb, Faculty of Agriculture (ZAGR).

In our analyses, we divided islands into five groups (sub-archipelagos) corresponding to their administrative location in five regions (županija): 1) Kvarner islands, 2) Zadar islands (North Dalmatian Islands), 3) Šibenik islands, 4) Split islands (Central Dalmatian Islands), and 5) Dubrovnik islands (South Dalmatian Islands).

## Results

We recorded the use of 146 taxa from 59 families and identified the use of 131 of them for at least one medicinal purpose (Table 2). The remaining 15 species were used as a “healthy herbal tea”. The frequency curve is quite steep and 43 of them were recorded only in one interview, and 15 in two interviews, and only 88 were recorded in more than two interviews. Out of all the archipelagos within the Adriatic islands, the largest number of taxa both in terms of the total number of species (88) and the mean number of species listed per interview was recorded in the Dubrovnik archipelago (Table 1). The lowest number of species per interview was recorded in Kvarner. The shortest list of species (50) was recorded in the Šibenik archipelago (probably due to the smaller area and number of islands as well as the smallest number of interviews). When we look at single islands, the longest list of medicinal plants used was recorded on Korčula (66 species), Lošinj (45), Mljet (41), Šolta (37), Lastovo (35) and Molat (31). The highest mean number of species listed (Table 1) was recorded on Prvić (8.8), Korčula (8.4) and Šipan (8.2).

**TABLE 2:** List of species used medicinally or as herbal tea in the study area.

Scientific names and voucher no	Kvarner	Zadar	Šibenik	Split	Dubrovnik	Total	Islands	Local names	1	2	Medicinal uses
*Achillea millefolium* L. (Asteraceae) WA72398	2		2		6	10	Korčula, Lastovo, Lošinj, Prvić	stolisnik, hajdučka trava, vačja trava	s/m	fl	Herbal infusion for stomach; ingredient in ointment against varicose veins
*Aesculus hippocastanum* L. (Sapindaceae) ZAGR44283	2					2	Cres, Lošinj	divlji maruni, divlji kesteni	s	sd	Soaked in rakija for smearing leg veins and against rheumatism
*Agrimonia eupatoria* L. (Rosaceae) WA66922					1	1	Korčula	[no name]	s	ae	Cream against hemorrhoids
*Ajuga chamaepitys* (L.) Schreb. (Lamiaceae) WA66388	3	6		3	1	13	Drvenik veli, Koločep, Korčula, Ilovik, Pašman, Silba, Šolta, Ugljan	trava iva, ivica, ivina trava	s/m	ae	Medicinal infusion, medicinal rakija spice, panacea
*Allium sativum* L. (Amaryllidaceae)	1					1	Cres	češnjak	s	wh	Olive leaf and garlic in rakija, 20–40 drops a day for lowering blood pressure
*Aloe vera* (L.) Burm.f. (Asphodelaceae)	4				1	5	Lastovo, Lošinj	aloja, aloj	s	l	Against burns and wounds, facial tonic
*Aloysia citriodora* Palau (Verbenaceae) WA66484			1	1	13	15	Korčula, Lastovo, Vis, Zlarin	limončelo biljka, alviz, alviža, alvižej, vervena, aluiza, bella luiga, bavliža, albaluija	a	ae	Infusion for stomach
*Althaea officinalis* L. (Malvaceae)	4	3	1			8	Dugi Otok, Iž, Krk, Lošinj, Prvić, Unije	bijeli slez, bijeli sljez, sliz	s	wh	Infusion for the gums, lungs, against colds and cough; root in water macerate for bronchia
*Anethum graveolens* L. (Apiaceae) WA66391				1		1	Drvenik Veli	kopar	s	ae, l	Infusion
*Arbutus unedo* L. (Ericaceae) WA66321		1		1		2	Molat, Šolta	planika, magunja	s	l	Infusion, against prostate problems, collected in May and dried, probably new knowledge
*Arctium lappa* L. (Asteraceae)		1				1	Pag	čičak	s	rt	Infusion against tumors, cleaning the blood and tonic for liver
*Artemisia abrotanum* L. (Asteraceae) WA72346					5	5	Lastovo, Korčula	srčano zelje, srčeno zelje, pelin, broda	s	ae	Infusion to induce menstruation
*Artemisia absinthium* L. (Asteraceae) WA66375	15	12	3	8	5	43	Throughout	pelin, asincio, ašinac, pitomi osjenač, pelen	s/m	ae	Infusion for digestion, stomach ache, worms, cancer; leaves pressed to wounds; medicinal rakija spice; applied on sprains with egg white, relieves pain and reduces swelling
*Artemisia caerulescens* L. (Asteraceae) WA66473	1					1	Cres	divlji pelin, pelino	s	ae	Infusion/rakija, also against worms
*Asparagus acutifolius* L. (Asparagaceae) WA66368	1	1	1	1	2	6	Dugi Otok, Korčula, Lošinj, Šolta, Žirje	šparoge	s	wh	Strengthens kidneys and promotes urination
*Asplenium trichomane*s L. (Aspleniaceae)					1	1	Šipan	paprat	s	ae	Infusion against kidney stones
*Ballota nigra* subsp. *foetida* (Vis) Hayek (Lamiaceae) WA72353					1	1	Korčula	saserina	s	ae	Compress (for children) for stomach aches caused by worms, and therefore for better appetite
*Bellis perennis* L. (Asteraceae) ZAGR55974	1					1	Rab	divlja tratinčica	s	ae	Medicinal rakija for massaging swollen legs
*Bituminaria bituminosa* (L.) C.H.Stirt. (Fabaceae) ZAGR46682	1				1	2	Cres, Korčula	djetelina velika	s	ae	Infusion for washing face, dried leaves given to sheep against diarrhea
*Borago officinalis* L. (Boraginaceae) WA72385					1	1	Lastovo	modri cvjet	s	ae	Medicinal infusion
*Brassica oleracea* L. ssp. *acephala* (Brassicaceae) ZAGR49468	2	1				3	Dugi Otok, Ilovik, Unije	broskva, raštika	s/m	l	Leaves pressed on hurting organs (to draw pain from head, knees etc.); warm leaf with olive oil placed on chest against bronchitis
*Calendula arvensis* (Vaill.) L. (Asteraceae) WA66425		1			2	3	Dugi Otok, Korčula	neven, divlji neven	s	fl	Cream for skin; rubbed into skin with oil
*Calendula officinalis* L. (Asteraceae) WA66413	4	4	3		1	12	Lošinj, Mljet, Molat, Prvić, Rab, Vrgada	neven	s/m	fl	Infusion (good for blood), oil, cream, ointment (esp. With home made lard) against vein inflammation, wounds, aching bones; medicinal rakija for massaging swollen legs
*Capparis orientalis* Veill. (Capparaceae) WA72302					1	1	Lopud	kapare	s	fl	Infusion
*Centaurium erythraea* Rafn (Gentianaceae) WA66924	7	6	1	2	2	18	Dugi Otok, Hvar, Korčula, Iž, Lošinj, Mljet, Molat, Prvić, Silba, Susak, Unije, Vrgada	kičica, gorčilo, semencina, muška kantarija, mužka gospina trava, centaurea	s	ae	Infusion for stomach, intestines, appetite; for children against fever; cancer
*Ceratonia siliqu*a L. (Fabaceae) WA66455		3		1	9	13	Dugi Otok, Korčula, Lastovo, Lopud, Mljet, Šipan, Šolta	rogač, harub, karuba	s/m	fr	Good raw for stomach against diarrhea; in a infusion mix with honey for cough
*Ceterach officinarum* Willd. (Aspleniaceae) WA72375		1		3	8	12	Brač, Hvar, Iž, Korčula, Lopud, Mljet, Šipan, Šolta	paprat, zlatin, oslad, zlatac, prapratac, divlja paprat	s/m	l	Infusion for internal organs eg. Liver, stomach, high pressure, bladder, jaundice, kidneys, bile flow; panacea (elixir of youth giving vis vitalis)
*Cichorium intybus* L. (Asteraceae) WA72358				1		1	Šolta	žutinica	s	ae	Decoction against diarrhea
*Cistus salviifolius* L. (Cistaceae) WA6649	2					2	Lošinj, Unije	bušin	s	l, fl	Infusion against gallstones, for leg veins
*Citrus × aurantium* L. (Rutaceae) WA71108				2	4	6	Koločep, Korčula, Šipan, Vis	ljuta naranča, divlja naranča, naranča	s/m	p, l	Infusion
*Citrus limon* (L.) Osbeck (Rutaceae) WA66464	1	1		2	1	5	Ilovik, Pašman, Šipan, Šolta, Vis	limun, limon	s/m	p, l, fr	Infusion against cough
*Clematis vitalba* L. (Ranunculaceae) WA66476	1					1	Cres	škrebut	s	ae	Macerated leaves as compress against rheumatism
*Clinopodium nepeta* (L.) Kuntze (Lamiaceae) WA66943	3	16	5	4	19	47	Throughout	metvica, divlja metica, ošja metvica, tovarska metvica, magareća menta, origano divlji, divlja menta	s	ae	Infusion for stomach, heart, cleaning blood, inducing appetite, against menstrual pains, bronchitis, colds and cough
*Cornus mas* L. (Cornaceae) WA66480	2					2	Lošinj	drijen, dren, drenjula	s	l, fl	Infusion
*Crataegus monogyna* Jacq. (Rosaceae) WA66444		3			2	5	Dugi Otok, Korčula, Lastovo, Pag, Silba	glog	s	fr, fl	Infusion, also good for the heart
*Cupressus sempervirens* L. (Cupressaceae)					1	1	Hvar	[no name] čempres	s	res	Resin to heal skin cracks
*Cydonia oblonga* Mill. (Rosaceae) WA72370					1	1	Korčula	dunja	s	l	Infusion against diarrhea
*Cymbalaria muralis* P.Gaertn., B.Mey. and Scherb. (Plantaginaceae) ZAGR56756			1			1	Prvić	[no name]	s	l	Fresh crushed leaves on wounds
*Cynara scolymus* L. (Asteraceae)				1		1	Vis	artičoka	s	l	The rib of a large leaf used to make infusion for liver
*Dioscorea communis* (L.) Caddick & Wilkin (Dioscoreaceae) ZAGR39307	2	1			1	4	Dugi Otok, Korčula, Krk	bljušć, blušt, kukelj	s	wh	Medicinal rakija against swelling legs and rheumatism; infusion against cough
*Diplotaxis tenuifolia* (L.) DC. (Brassicaceae) WA72350		1				1	Pašman	divlja riga	s	l	Infusion
*Ecballium elaterium* (L.) A.Rich. (Cucurbitaceae) WA66901	1	2	2		1	6	Krapanj, Lastovo, Pašman, Susak	štrokalica, zlatin, divlji krastavac, šćukavica	s	fr	Sap from fruits applied in the nose against sinusitis
*Elymus repens* (L.) Gould (Poaceae) WA71140	4	1	2			7	Krk, Lošinj, Pašman, Prvić, Silba, Susak	pirika, pirac, perika, troskot, truoskut	m	u	Infusion and rakija for cleaning the organism and urinary tract, used esp. For bladder problems and prostate; compress against styes
*Equisetum* spp. (Equisetaceae) (*E. ramosissimum* Desf. WA66939, *E. telmateia* Ehrh WA66482)	1	2				3	Pag, Rab	nadudäč, konjski rip, nadudavnjak	s	ae	Infusion for urinary tract and kidneys
*Erica arborea* L. (Ericaceae) WA66432	1	2		6	3	12	Hvar, Korčula, Mljet, Pašman, Šolta, Unije, Vis, Vrgada	vris, vrjes, vris bili, vrisak, vrjesak, vrijesak	m	ae	Infusion for bladder and prostate
*Erica manipuliflora* Salisb. (Ericaceae) WA66301				1	2	3	Korčula, Šolta	vrisak, vrisac, vrjesak	s	ae	Infusion
*Eucalyptus globulus* Labill. (Myrtaceae) ZAGR49486	3					3	Lošinj	eukaliptus	s/m	l	Inhalations and infusion for respiratory system
*Euphorbia characias* L. subsp. *wulfenii* (Hoppe ex W.D.J. Koch) Radcl.-Sm. (Euphorbiaceae) WA72287		1			1	2	Dugi Otok, Mljet	lumbrosik, mljekocvjet	s	s	Sap externally for wounds and against warts
*Ficus carica* L. (Moraceae) WA72367	1	2		1	2	6	Cres, Dugi Otok, Korčula, Mljet, Pag, Vis	smokva	s/m	fr,l	Infusion against colds, for prostate and digestion
*Foeniculum vulgare* Mill. (Apiaceae) WA66401	4	10	7	6	6	33	Throughout	koromač, komorač, kromač, morač, divlji koromač	m/s	ae, fr	Infusion for digestion, against cold and arthritis, detoxifying
*Geranium macrorrhizum* L. (Geraniaceae) ZAGR56752					1	1	Korčula	kanela	s	l	Infusion
*Hedera helix* L. (Araliaceae) WA72316					3	3	Korčula, Mljet	bršćan, brštan, bršljan	s	ae	Cream for skin against eczema; infusion externally for washing hemorrhoids and strengthening hair
*Helichrysum italicum* (Roth) G.Don (Asteraceae) WA66460	5	15	2	3	4	29	Throughout	magriž, marzih, smilje, smilj, cmij	s	ae	Essential oil for cosmetics (new uses); infusion esp. For diabetes and allergies (probably new uses); dry branches were burned in the fireplace in the house as disinfectant
*Hyoscyamus albus* L. (Solanaceae)			3			3	Krapanj, Prvić	bunika	s	l	Compress for ulcers and wounds
*Hypericum perforatum* L. (Hypericaceae) WA66461	16	44	14	24	22	120	Throughout	gospina trava, gospino cviće, gospina trova, gospina dušica, kantarion, cvijet sv. ante, cvijet sv. Ivana, ivanijsko cvečje, ulinjak	s	ae, fl	“Kantarion” oil (macerate made by steeping fresh flowers in olive oil for 40 days in the sun) against rheumatism and wounds applied externally; infusion used for stomach and against depression (the latter is probably a new use)
*Inula helenium* L. (Asteraceae)	1					1	Lošinj	oman	s	u	Medical infusion (panacea)
*Inula verbascifolia* (Willd.) Hausskn. (Asteraceae) WA66432				1		1	Brač	bilušina	s/m	ae	Compress for stomach ache in children
*Jasminum officinale* L. (Oleaceae)			1			1	Žirje	jasmin	s	fl, l	Infusion
*Juglans regia* L. (Juglandaceae) WA72310		3	3	1		7	Brač, Prvić, Ugljan, Vrgada, Zlarin, Žirje	orah, orih	s	im, l	Rakija or liqueur for stomach; young leaves as infusion against diabetes and constipation
*Juniperus macrocarpa* Sm. (Cupressaceae) WA66907, and *J. oxycedrus* L. WA66332		1		4	5	10	Brač, Dugi Otok, Hvar, Korčula, Vis	smrička, smrič, smriča, smrkinja, smreška, pukovnica, klekinja, badljač	s	fr, ae	Infusion for blood cleaning, prostate, against flu
*Laurus nobilis* L. (Lauraceae) WA66310	8		3	4	2	17	Brač, Cres, Krapanj, Lošinj, Mljet, Rab, Susak, Šolta, Žirje	lovor, lovorika, lumber, laurano, javor, javorika	s/m	l, fr	Infusion or decoction from leaves against diarrhea. And cough; oil (“lumberovo ulje”) for smearing the belly button and temples to get rid of worms, also used against carnuncles; fruits syrup against cough
*Lavandula x intermedia* Emeric ex Loisel. (Lamiaceae) WA71147, and *L. angustifolia* Mill. WA66937	1	1	2	2	5	11	Kaprije, Korčula, Krapanj, Lastovo, Pag, Lošinj, Šolta, Vrgada	lavanda, levanda, lavandur, lavandin, petašenca, pitašencija	s	l, fl	Infusion and essential oil for easier sleep; lavender oil used to smear belly button, *dešpik* (lavender flowers in vinegar) was used against worms
*Lavatera arborea* L. (Malvaceae) ZAGR56750	2	1		1	4	8	Ilovik, Korčula, Mljet, Molat, Šolta	veliki slez, veliki sljez, veliki sliz, bili sliz, bijeli sljez, bijeli cliz	s	l, fl	Infusion against colds and cough
*Lilium candidum* L. (Liliaceae) WA71156	1	1	2		1	5	Dugi Otok, Ilovik, Korčula, Zlarin, Žirje	bijeli ljiljan, ljiljan, lilian, cviće sv. antuna	s/m	fl	Oil macerate applied to wounded or bruised skin
*Malva sylvestris* L. (Malvaceae) ZAGR49704, and *M. neglecta* Wallr. ZAGR39610	13	24	3	12	26	78	Throughout	sljez, sliz, slez, crni sljez, Mali sljez, divlji sljez, malva	s	wh	Infusion against cough, bronchitis, for throat, lungs and urinary tract
*Marrubium peregrinum* L. (Lamiaceae) WA72308					1	1	Korčula	trava od strahe	s	ae	Place into socks overnight for children’s fright
*Matricaria chamomilla* L. (Asteraceae) WA66467	16	42	18	26	17	119	Throughout	kamilica, kamomila, kamumil, kamilia, kamila, divlja kamamilica, kamonela, kalomela, kalumela	s	fl	Infusion, mainly calming, also against constipation, menstrual pain; infusion on cotton placed in ear against ear pain
*Melissa officinalis* L. (Lamiaceae) WA66495	2	6	3	3	16	30	Throughout	melisa, matičnjak, pčelina ljubica, čelina jubica, jubica	s	ae	Mainly calming infusion, also cream for skin against herpes
*Mentha* spp. (Lamiaceae) (*M. spicata* L. WA66348, and *M.* x *piperita* L. WA66402)	7	12	8	10	34	71	Throughout	menta, metvica, metica, divlja metica, burgameta, domaća metvica, paprena metvica	s/m	ae	Infusion against gastrointestinal problems or diabetes, for calming
*Morus nigra* L. (Moraceae) ZAGR39664, and *M. alba* L		1	1	3		5	Brač, Hvar, Molat, Prvić	murva (dud), murva bila (bijeli dud), murva crna (crni dud)	s	l	Young leaves as infusion against diabetes, lowering sugar level (new use)
*Myrtus communis* L. (Myrtaceae) WA66307	3	1		1	3	8	Lopud, Lošinj, Mljet, Molat, Rab, Šipan, Šolta, Unije	mirta, mrča, mrta, mrčika, marta, marka	s	fr	Raw and infusion for stomach or thyroid; infusion and tincture with pork lard traditionally used against hemorrhoids
*Nicotiana* sp. (Solanaceae)	1					1	Unije	divlji duhan	s	l	For children: Put on the chest to breathe more easily
*Ocimum basilicum* L. (Lamiaceae) WA71155		2	4		3	9	Kaprije, Korčula, Krapanj, Pašman, Silba, Zlarin, Žirje	bosiljak, divlji bosiljak, murtela	s	ae	Infusion, also for washing hair; a leaf kept behind waist as perfume
*Olea europaea* L. (Oleaceae) WA72282	8	6	2	3	2	21	Throughout	maslina	s	l, fr	Infusion from leaf for lowering and/or equalizing blood pressure; macerated leaf of olive and garlic in rakija, 20–40 drops a day for lowering pressure; young leaves as anti-diabetic infusion; in rakija for digestion; heated olive oil drops put into ear against ear pain
*Origanum majorana* L. (Lamiaceae) WA66443		2	2	2	19	25	Throughout	mažuran, mažurana, mažuranka, mežuran, mizorana, sansek	s/m	ae	Infusion, essential oil
*Origanum vulgare* s.l. (Lamiaceae) WA71146		2	3	3	8	16	Throughout	origano, divlje origano, mravinac, mravjenac	s/m	ae	Infusion for stomach and against headache
*Paliurus spina-christi* Mill. (Rhamnaceae) WA72298	1	7	6	2	10	26	Throughout	drač, drača, crna drača, isusovo trnje, trn, šeširići, dinari, botuni, puco od trnja	s	im, fr	Infusion against diarrhea., kidney stones, hemorrhoids and gout
*Parietaria judaica* L. (Urticaceae) WA66338	17	9		3	4	33	Throughout, esp. in Kvarner	šćir, šćirenica, šćurenica, šćinjerica, škinjerica, ščavec, ščirpenica, crkvina, odrimak, lepek, drenak	s	ae	Infusion mainly for kidneys, prostate and bladder, also veins and gynecological diseases; compress for bruises; also used to heal chicken pip (psittacosis) – used to make balls of *P. judaica* with olive oil to treat this ailment
*Passiflora caerulea* L. (Passifloraceae) WA66498					1	1	Mljet	gospina kruna	m	fl	Infusion
*Pelargonium odoratissimum* (L.) L'Hér. (Geraniaceae) WA71132					2	2	Korčula	barbaroza, barbaška	m	l	Infusion
*Petroselinum crispum* (Mill.) Fuss (Apiaceae)					1	1	Korčula	petrusin	m	l	Infusion against cough
*Pimpinella anisum* L. (Apiaceae)					2	2	Korčula, Mljet	anis, aniš, aniž	s/m	fr	Infusion
*Pinus halepensis* Mill. (Pinaceae) WA71148	1	2		1	1	5	Hvar, Ilovik, Korčula, Lošinj, Molat, Vis, Vrgada	bor alepski, bor	s, cn	l	Mainly syrup against cough, asthma and other respiratory problems, made from young shoots and unripe cones, also good for kidneys
*Pinus nigra* J.F.Arnol (Pinaceae) WA66899	2					2	Cres, Lošinj	bor	s	l	Syrup from young shoots used against cough – a new fashion from the continent
*Pistacia lentiscus* L. (Anacardiaceae) WA66383	1			1		2	Šolta, Unije	lonjstik, smrča	s	l, fr	Leaf macerate with vinegar against scratches, bruises; fruit macerated in rakija smeared on the chest for the thyroid gland
*Plantago* spp. (Plantaginaceae) (*P. lanceolata* L. WA71162, *P. major* L. ZAGR39699, and *P. media* L. ZAGR39712)	3	9	1	5	10	28	Throughout	trputac, trputac ženski, trputac muški, bokvica, bukvica, pasji jezik, pasi jazik	s	l	Syrup from leaves buried for 3 months; syrup stays bottled in the ground for 45 days with a layer of sugar and layer of leaves; infusion against cough, bronchitis and for lungs; raw placed on wounds, needs to be peeled from hairs first; juice from leaves against ear pain and warts
*Plumbago europaea* L. (Plumbaginaceae) WA66914					1	1	Korčula	vranjomil	s	l	Compress against wounds and bruises
*Portulaca oleracea* L. (Portulacaceae) WA66314		1				1	Ugljan	tušć	s	l	For wounds
*Prunus cerasifera* Ehrh. (Rosaceae) WA66497	1	1				2	Molat, Unije	armelin, slivić, sužin	s	fr	Raw against constipation
*Prunus spinosa* L. (Rosaceae) WA66367				1	2	3	Brač, Lastovo	divlji trn, trn, trnina	s	fr	Infusion, good for heart
*Prunus dulcis* (Mill.) D.A.Webb (Rosaceae)	1	5		3	6	15	Brač, Dugi Otok, Korčula, Lastovo, Lošinj, Mljet, Pag, Pašman, Šipan, Šolta	badem, bajam, mendel, mindel, mjendul	s/m	pe	Infusion for bladder, prostate; infusion for kidneys; against cold and cough; against diabetes; almond peel, carob and dried figs cooked to cleanse the respiratory organs; anti-cough syrup
*Punica granatum* L. (Lythraceae) WA66478	6	3	1		3	13	Throughout	mogranj, mugranj, mogronj, nar, divlji šipak	s	pe	Infusion aganst diarrhea
*Pyracantha coccinea* M.Roem. (Rosaceae) WA66898	1					1	Cres	pasji trn, spina de kan	s	fr	Fruit good for illnesses of respiratory system, gives energy when eaten
*Pyrus amygdaliformis* Vill. (Rosaceae) WA66904	1					1	Rab	divlja kruškica, grogulja	s	fr	Raw and dried in small amounts against diarrhea
*Quercus ilex* L. (Fagaceae) WA72378	1	1				2	Cres, Zverinac	hrast crnika, crnika	s	l	Washing genitals and wounds, leaves given to sheep against diarrhea
*Quercus pubescens* Willd. (Fagaceae)					1	1	Korčula	hrast medunac, dub	s	wood	To cure fright: Burn the branch, put hot oak coal in wine for the night, drink in the morning on empty stomach
*Reichardia picroides* (L.) Roth (Asteraceae) WA66427	1			1		2	Lošinj, Šolta	jagla, dušica	s	ae	Decoction for purifying the blood
*Robinia pseudoacacia* L. (Fabaceae) WA66466		5	1			6	Molat, Pag, Silba, Žirje	bagrem, akacija, drača	s	fr	Infusion against asthma
*Rosa* spp. (Rosaceae) (*R*. *canina* L. WA66309, and *R. sempervirens* L. WA66323)	36	29	6	34	14	119	Throughout	divlji šipak, šipak, šipurika, šepun, šipunić, šepurika, roza selvatika, štropakul, divlji lunzor, divjo ruža, svrbiguzica, šošić, šušić	s/m	fr	Mainly infusion, often for diarrhea
*Rosmarinus officinalis* L. (Lamiaceae) WA66366	1	2	6	5	9	23	Throughout	ružmarin, lucmarin, rusmarin, ruzmarin, zoromod, zemorod	s	l	Essential oil against respiratory problems or headache and for massage, externally against rheumatism (for the knees), perfume; syrup against cough; infusion for circulation
*Rubia peregrina* L. (Rubiaceae) WA72327					1	1	Mljet	broć	s	u	Infusion for kidneys
*Rubus ulmifolius* Schott (Rosaceae) WA72295	4	4	4	2	5	19	Throughout	divlja kupina, kupina, kumpjena, jagoda, ostruga, zrača	s	fr, l, fl, rt	Infusion as antidiarrhea.l agent, for rinsing gums, against bronchitis and to promote urination
*Rumex crispus* L. (Polygonaceae)	1					1	Unije	konjski štavelj	m	l	Against diarrhea
*Rumex pulcher* L. (Polygonaceae) ZAGR39692		2		1	1	4	Mljet, Pašman, Šolta, Ugljan	štavelj, štavljak, čovljak	s	ae, fl, fr	Decoction from seeds or flowers against diarrhea
*Ruscus aculeatus* L. (Asparagaceae) WA66369			1			1	Prvić	leprinac	s	ae, rt	Medicinal infusion
*Ruta graveolens* L. (Rutaceae) WA66380	4	8	2	2	11	27	Throughout	ruta, rutva, rutvica	s/m	ae	Medicinal rakija for stomach, lack of appetite, against worms, abortive, to cure infertility; leaves and their juice as medicine to cure children’s lack of appetite; to protect from the evil eye – when a child was frightened, it was fried in oil and put on a cloth on the child’s stomach
*Salvia officinalis* L. (Lamiaceae) WA66559	38	68	21	49	37	213	Throughout	kadulja, kaduja, slavulja, slavuja, kuš, pelin, žalfija	s/m	l, fl	Infusion or decocted with milk against throatache, various kinds of respiratory problems, against diorrhoea; fresh twig chewed against toothache and mouth infections
*Sambucus nigra* L. (Adoxaceae) WA66415	4	4		6	13	27	Hvar, Korčula, Lastovo, Lošinj, Mljet, Molat, Silba, Šolta, Unije	bazga, sambuk, štambuk, baz, bazgovina	s	l, fl	Mainly flowers for infusion to cure respiratory problems and infections, also for bladder and infusion from young leaves for cleaning blood
*Santolina chamaecyparissus* L. (Asteraceae) WA71111				1		1	Vis	trovo od crvih	s	ae	Macerate in rakija as vermifuge
*Satureja montana* L. (Lamiaceae) WA66397	5	7	4	12	6	34	Throughout	vrisak, vris, tovarski vris, kidež, bresina	s	ae	Infusion good for urinary tract and circulation, against common cold; macerate in rakija against hair loss
*Satureja visianii* Šilić (Lamiaceae) WA66385					2	2	Korčula	vrisak mali	s	ae	Infusion
*Sedum* cf *telephium* L. s.l. (Crassulaceae) WA66494	2		3	1	4	10	Korčula, Lastovo, LoŠinj, Mljet, Prvić, Rab, Šolta	bobovnik, bobovnjak, tusanj, tušć	s	l	Squashed leaves as compress on wounds and ulcers, against ear pain, as ulcer dressing; wrapped leaf against backache
*Sempervivum tectorum* L. (Crassulaceae)	7	8	2	1	1	19	Throughout	čuvarkuća, pazi kuća	s	s	Sap from squashed leaves against earache and warts
*Silene vulgaris* (Moench) Garcke (Caryophyllaceae) WA71139		1				1	Vrgada	(no name)	s	l	Infusion for strengthening knees
*Silybum marianum* (L.) Gaertn. (Asteraceae) WA66349		7		2		9	Hvar, Ist, Molat, Pag, Pašman, Ugljan, Vis	sikavica, sikavac, badič, ošćibod	s	ae	Infusion for cleaning liver, kidneys, very good for regulation of sugar level, regulation of high pressure; macerate in rakija as a stomach remedy (all uses probably highly influenced by literature)
*Sorbus domestica* L. (Rosaceae) WA66319	3	1		4	7	15	Throughout	oskoruša, skoruša, skorušva, škoršva, oksoruša	s	fr	Eaten raw or dried against dysentery and diarrhea. (Put in hay to soften), dried in necklaces made of half-cut fruits
*Stachys cretica* subsp. *salviifolia* (Ten.) Rech.f. (Lamiaceae) WA722297					1	1	Korčula	zečje uho	s	l	Formerly for dressing wounds
*Symphytum* cf *tuberosum* L. (Boraginaceae)					1	1	Mljet	gaviez, gavez	s	rt	Macerate or ointment to heal broken bones
*Tanacetum balsamita* L. (Asteraceae) WA66414			2		2	4	Korčula, Krapanj, Zlarin	kaloper	s	l	Infusion, also used as perfume placed behind ears or tied to the head
*Taraxacum* sp. (Asteraceae) WA66372	1		1		1	3	Cres, Korčula, Zlarin	maslačak, divlji radić, kostrič	s	fl	Infusion/syrup for immunity, macerate in olive oil for skin
*Teucrium chamaedrys* L. (Lamiaceae) WA72402			1			1	Prvić	dubčac	s	ae	Infusion against diabetes
*Teucrium montanum* L. (Lamiaceae) WA72403	3	3	1		2	9	Korčula, Krk	trava iva	s	ae	Medicinal infusion used as panacea – “to clean the body”, also for common cold
*Teucrium polium* L. (Lamiaceae) WA66373		1				1	Vrgada	trava iva	s	ae	Infusion
*Thymus* spp. (Lamiaceae) (Mainly *T. longicaulis* WA71151)	20	21	3	8	22	74	Throughout	majčina dušica, popunac, poponac, papunac, Timo	s/m	ae	Infusion for respiratory problems and common cold
*Thymus vulgaris* L. (Lamiaceae)					1	1	Mljet	timijan	s	ae	Infusion
*Tilia* spp. (Malvaceae) (*T. cordata* Mill. ZAGR39719, *T. platyphyllos* Scop. WA66300, and *T. tomentosa* Moench ZAGR39718)	9	3	1	5	9	27	Throughout	lipa, lipica	s	fl	Infusion against respiratory and heart problems
*Tordylium apulum* L. (Apiaceae) WA72329					1	1	Korčula	lembrc, limberc, vratimuža	m/s	fl, l	Infusion; also chewed to refresh breath
*Urtica* spp. (Urticacaeae) (*U. dioica* L. WA66401, *U. pilulifera* L. WA66441, and *U. urens* L. WA66423)	13	11	7	12	11	54	Throughout	kopriva, koprva, koprjeva, pokriva, ožigavica, užigavica, žigavica, užgalina, pruda	s/m	ae, l	Infusion as panacea, diuretic, against blood lipids, for kidneys, prostate, bladder, rheumatism, to wash hair to make it grow; fresh nettles used to sting on purpose against lumbago and sciatica
*Verbascum* sp. (Scrophulariaceae)	1					1	Lošinj	divizma	s	l	Syrup against cough
*Viola odorata* L. (Violaceae) ZAGR39660	1					1	Lošinj	ljubičica	s	fl	Infusion
*Vitex agnus-castus* L. (Lamiaceae) WA66303		3			1	4	Dugi Otok, Korčula, Pag	konopljika, konoplika	m	l, fl	Infusion for kidneys; attached to clothes to cure “fright” illness
*Vitis* cf *vinifera* L. (Vitaceae) ZAGR56758	1		2			3	Prvić, Susak, Unije	vinova loza, trs, grožđe (fruit)	s	fr, sd	Young leaves as infusion against diabetes; homemade rakija as panacea or disinfectant; rubbed on the body to lower temperature during infections; grape seeds against diarrhea; used to cure fright in children
An unidentified species – the name is usually applied to toxic species (*Veratrum, Helleborus, Colchicum*) but also to *Blackstonia perfoliata* and *Centaurium erythraea*)					2	2	Lastovo, Mljet	čemerika	s	l	Decoction to cure kidney stones and urine tract

1. s – single species, m –mixed species

2. Part used: ae – aerial parts, cn – cones, fj – fruit juice, fl – flowers, fr – fruit, im – immature fruits, l – leaves, p – peel, pe – pericarp, res – resin, rt – roots, s – cell sap, sd – seed, u – underground parts, wh – whole plant.

Most collected parts are aerial parts (flowering shoots with leaves or just leaves - 38% of use reports, flowers - 18%, fruits - 15%, underground organs - 0.8%).

A large majority of plants are collected from the wild (77%). As many as 66% are native to the flora and 13% are alien species. As many as 44% of the collected species are cultivated, so there is a large overlap between the two categories of wild and cultivated (e.g., *Salvia offcinalis, Ruta graveolens,* etc.).

**FIGURE 2:**
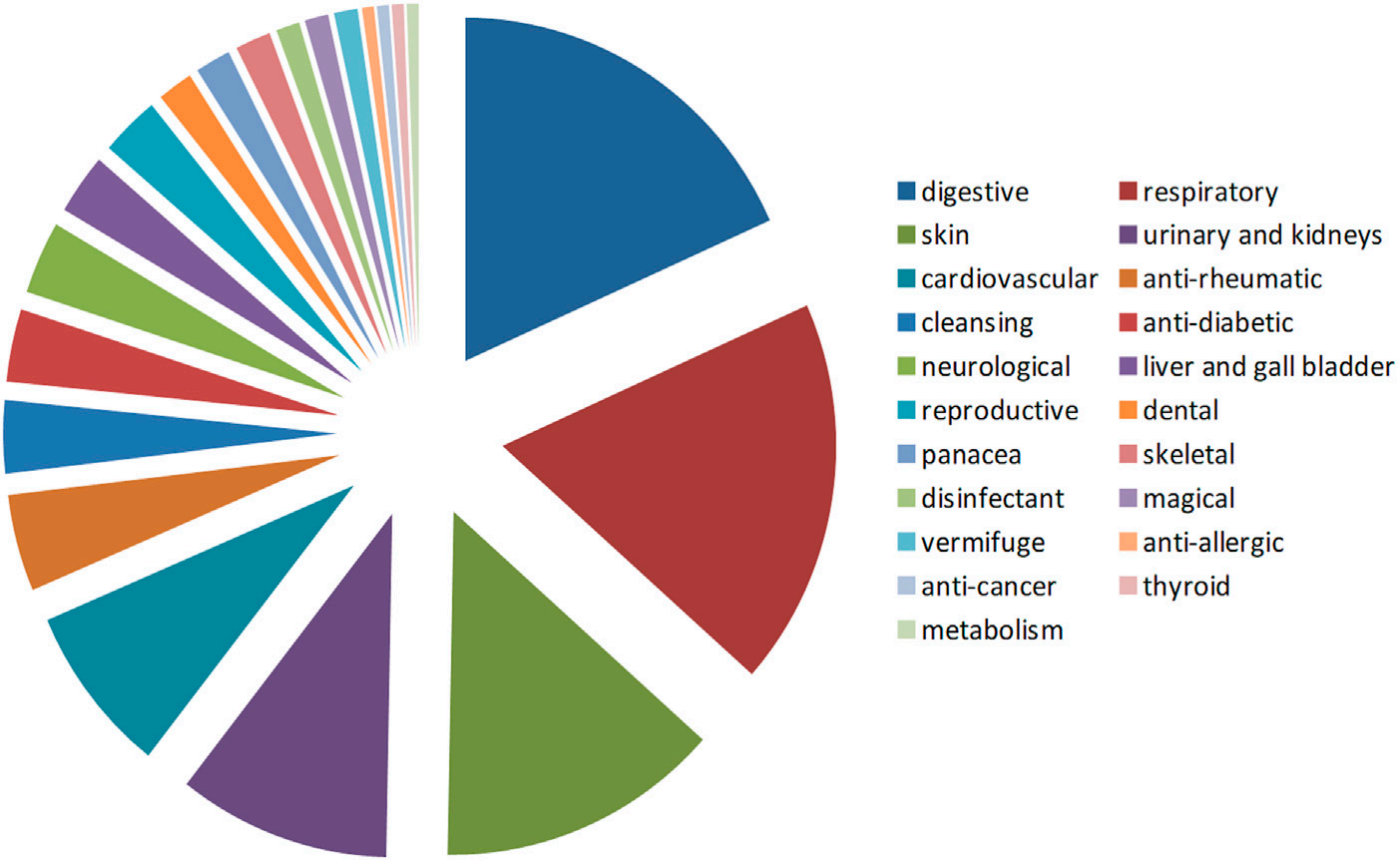
Distribution of ailments treated vs. plant species used.

Infusion is the most common form of herb use. Decoction and steeping in rakija is rarely used. *Rakija* is the local term for spirits distilled from fruits (mainly grapes in this study area). The most commonly cured ailments are digestive (18.3%), respiratory (13.7%) and skin (11.7%) problems. These three types constituted half of all the use reports (Figure 2).

The most commonly used medicinal plants in the area are: *Hypericum perforatum, Salvia officinalis, Parietaria judaica, Urtica* spp.*, Artemisia absinthium, Plantago* spp.*, Helichrysum italicum, Malva* spp., *Sempervivum tectorum* and *Matricaria chamomilla* (Table 3)*.* When cultural importance was expressed by the Use Value index, the species with highest score were: *Salvia officinalis, Hypericum perforatum, Rosa* spp.*, Matricaria chamomilla, Malva* spp.*, Thymus* spp.*, Mentha* spp.*, Urtica* spp.*, Clinopodium nepeta* and *Artemisia absinthium* (also Table 3).

**TABLE 3:** The most frequently used medicinal plants in the study area and the plants with the highest use value.

Species with highest frequency	No. of interviews with a clearly indicated medical use	Species with highest UV	UV
*Hypericum perforatum*	85	*Salvia officinalis*	0.117745
*Salvia officinalis*	57	*Hypericum perforatum*	0.066335
*Parietaria judaica*	30	*Rosa* spp.	0.065782
*Urtica* spp.	28	*Matricaria chamomilla*	0.065782
*Artemisia absinthium*	23	*Malva sylvestris* and *neglecta*	0.043118
*Plantago* spp.	22	*Thymus longicaulis*	0.040907
*Helichrysum italicum*	20	*Mentha* spp.	0.039248
*Malva* spp.	20	*Urtica* spp.	0.029851
*Sempervivum tectorum*	19	*Clinopodium nepeta*	0.025981
*Matricaria chamomilla*	18	*Artemisia absinthium*	0.02377
*Olea europaea*	18	*Satureja montana*	0.018795
*Laurus nobilis*	16	*Parietaria judaica*	0.018242
*Paliurus spina-christi*	16	*Foeniculum vulgare*	0.018242
*Ruta graveolens*	14	*Melissa officinalis*	0.016584
*Centaurium erythraea*	13	*Helichrysum italicum*	0.016031
*Sorbus domestica*	13	*Plantago* spp.	0.015478
*Foeniculum vulgare*	12	*Ruta graveolens*	0.014925
*Ceterach officinarum*	10	*Sambucus nigra*	0.014925
*Prunus amygdalus*	10	*Tilia* spp.	0.014925
*Rosmarinus officinalis*	10	*Paliurus spina-christi*	0.014373
*Sambucus nigra*	10	*Origanum majorana*	0.01382
*Thymus* spp.	10	*Rosmarinus officinalis*	0.012714

Most of the common species are used throughout the study area, from the Kvarner to the Dubrovnik Archipelago. If we look at correlations between island’s geographical longitude and proportion of interviews in which a given species is listed on the island, only a few species yield positive correlation indicating a tendency to be used more in the eastern part of the study area. The strongest such correlation was shown for *Ceterach officinarum, Sorbus domestica* and *Paliurus spina-christi* (Table 4)*.* There were no significant negative correlations, which would indicate grouping uses in the western part of the study area.

**TABLE 4:** Correlation between the island’s geographical longitude and the species frequency (on a scale of 0–1) on particular islands.

Species	*r*	*p*
*Ceterach officinarum*	0.61	***
*Sorbus domestica*	0.53	***
*Paliurus spina-christi*	0.51	**
*Ceratonia siliqua*	0.50	**
*Mentha* spp.	0.49	**
*Clinopodium nepeta*	0.41	*
*Prunus amygdalus*	0.40	*
*Melissa officinalis*	0.39	*
*Origanum vulgare* s.l	0.38	*
*Rosmarinus officinalis*	0.30	(*)
*Juniperus oxycedrus* and *J. macrocarpa*	0.29	(*)
*Plantago* spp.	0.26	ns
*Aloysia citriodora*	0.25	ns
*Origanum majorana*	0.18	ns
*Foeniculum vulgare*	0.18	ns
*Ruta graveolens*	0.15	ns
*Rosa* spp.	0.13	ns
*Sambucus nigra*	0.11	ns
*Lavandula* spp.	0.11	ns
*Achillea millefolium*	0.10	ns
*Erica arborea*	0.09	ns
*Sedum telephium* s.l	0.08	ns
*Ajuga chamaepitys*	0.05	ns
*Rubus ulmifolius*	0.04	ns
*Satureja montana*	0.04	ns
*Urtica* spp.	0.04	ns
*Malva sylestris* and *M. neglecta*	0.04	ns
*Olea europaea*	0.02	ns
*Tilia* spp.	0.02	ns
*Salvia officinalis*	-0.03	ns
*Thymus longicaulis*	-0.03	ns
*Punica granatum*	-0.05	ns
*Laurus nobilis*	-0.07	ns
*Calendula officinalis*	-0.07	ns
*Hypericum perforatum*	-0.11	ns
*Matricaria chamomilla*	-0.11	ns
*Sempervivum tectorum*	-0.17	ns
*Artemisia absinthium*	-0.24	ns
*Helichrysum italicum*	-0.26	ns
*Centaurium erythraea*	-0.28	ns
*Parietaria judaica*	-0.32	(*)

Statistical significance: ns – not significant (*) – not significant but approaching significance level (0.05 < *p* < 0.1), * <0.05, ** <0.01, *** <0.001.

### Multivariate Analysis and Correlations

**FIGURE 3:**
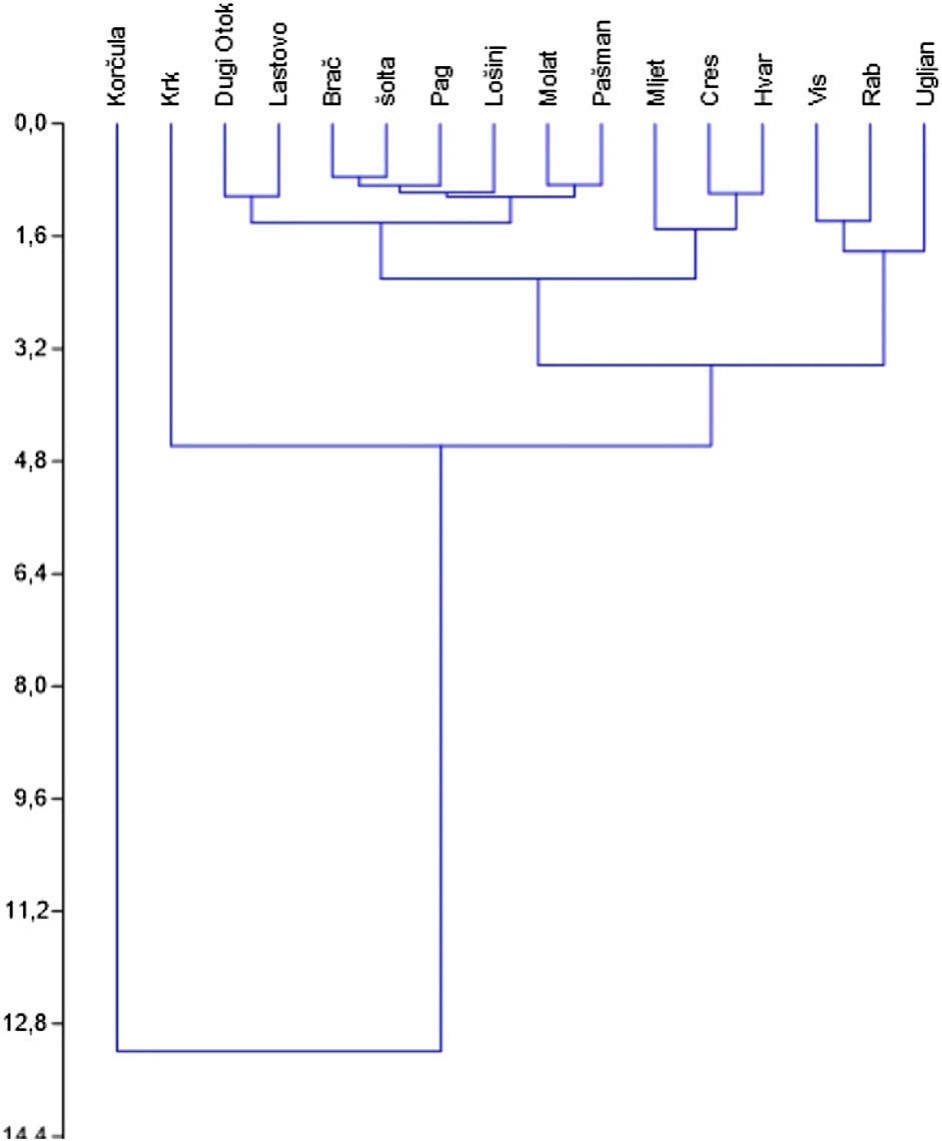
Dendrogram showing UPGMA clustering (with Euclidean distance) of islands (those where over 10 interviews were carried out).

**FIGURE 4:**
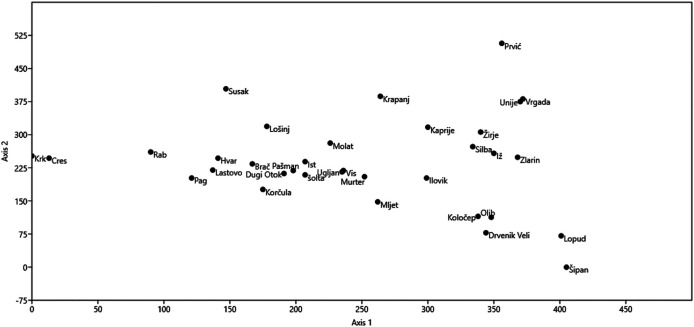
Detrended Correspondence Analysis biplot illustrating the two main axes of variability in the medicinal plant lists used in the studied islands.

Korčula is singled out in cluster analysis as the most dissimilar to other islands (Figure 3). In DCA analysis islands from the same sub-archipelago tend to be grouped close to each other (Figure 4), especially in the case of Kvarner. We did not find any significant correlations (Table 5) between the number of medicinal plants listed per island or mean number of species listed in an interview per island with geographical variables (size of island, population, longitude, number of species in the flora). Thus all of the hypotheses presented in the introduction can be rejected. The largest correlation (but still not significant) was between the total number of species used on an island and its population (r = 0.35), number of species in the flora of the island (0.34) and the area (0.29). For mean number of species per interview the largest correlation was longitude (0.28).

**TABLE 5:** Correlation matrix of the variables studied (data for 29 islands, those for which the number of species in their flora is known). Left-bottom corner – correlation coefficient *r,* Right-top corner *p* values. Correlation matrix for the studied variables.

0.00	Total no of species used	Mean no of species used	Area	Population	Flora	Longitude	Isolation
Total no of species used	0.00	0.90	0.13	0.07	0.07	0.50	0.93
Mean no of species used	−0.03	0.00	0.18	0.57	0.89	0.15	0.50
Area	0.29	−0.26	0.00	6.5E-08	1.5E-05	0.74	0.16
Population	0.35	−0.11	0.82	0	5.9E-05	0.83	0.15
Flora	0.34	0.03	0.71	0.68	0.00	0.34	0.87
Longitude	0.13	0.28	−0.06	−0.04	−0.18	0.00	0.05
Isolation	0.02	−0.13	−0.26	−0.28	−0.03	−0.37	0.00

## Discussion

### Some More Interesting Plant Uses

*Aloysia citriodora* is commonly used on Korčula as a recreational herbal tea and as a stomachic; it is much less known outside this island. According to a local legend, it was brought by a sailor from South America a long time ago and people shared the plant with each other.

*Artemisia caerulescens* is a rare coastal absinth species which was highly prized on Cres for aromatic liqueur-making. The tradition has now nearly ceased due to the disappearance of the plant, whose habitats were destroyed by the tourism industry and which has been overharvested.

Bay (*Laurus nobilis*) is a spice and medicinal plant commonly used throughout the coast of Croatia. It is usually the leaves that are used; however, on Cres and Rab, there is a tradition of making a syrup from its fruits and using it to fight coughs as well as of using bay oil (“lumberovo ulje”) medicinally, especially as an externally applied vermifuge.

Two species of horsetail rarely used in Europe, *Equisetum ramosissimum* and *E. telmateia*, are used medicinally on Rab and Pag.

*Satureja visianii* is an endemic savory species restricted to Pelješac Peninsula and Korčula Island. It is included in herbal teas on par with *Satureja montana*.

### Importance of a 40-day Period

An interesting phenomenon in the Adriatic folk pharmacopoeia is the importance of the period of 40 days for the maceration of plants in olive oil or keeping them in rakija. This is especially the case for “kantarion” *Hypericum* ointment for skin problems. The number of days was also mentioned e.g. on the occasion of making *Plantago* cough syrup or herbal liqueurs. The number 40 may have been chosen because of its importance in the Catholic liturgical year-Ascension Day comes forty days after Easter and Lent also lasts forty days; children are traditionally christened 40 days after birth (Đapo, 2015). Also quarantine, which was invented by the Republic of Ragusa (city Dubrovnik) for its visitors and lasted 30 days, and was later adopted by other circum-Mediterranean cities, especially by the Venetians, was extended to last 40 days until ship crew were allowed in a city (Anonymous, 2013).

### Trava Iva

In Croatia “trava iva” is primarily the name of *Teucrium montanum*, a plant highly prized along most of the Croatian coast (Šugar, 2008). There are a number of sayings about this plant (e.g., “trava iva (od) mrtva čini/pravi živa”-it “brings the dead to life”) which illustrate its nature of panacea. On the islands *Teucrium montanum* is rare, and two other species are named *trava iva* and given the same powers. These are *Ajuga chamepytis* in the Zadar Archipelago and *Teucrium polium* (throughout). *Iva* is an ancient plant name in southeastern Europe. It was also used to name the closely related *Ajuga iva* (L.) Schreb. *Iva/iwa* describes various plants in different European languages, e.g. *iwa* for *Salix caprea* in Polish and continental Croatian, for *Taxus baccata* in German, and Latvian ī*we* also for *T. baccata* (Brückner, 1927), *ivy* for *Hedera helix* and *ground ivy* for *Glechoma hederacea* in English, and the Latin name *Iva* for a plant from the Ambrosiaceae family.

### Fright

On the island of Korčula the local use of medicinal plants preserved some traditions of curing folk illnesses classified as “fright”- situations when a child would get frightened, cry a lot and become restless-using *Marrubium peregrinum, Ruta graveolens, Vitex agnus-castus* or charcoal oak. The illness is known in folk pharmacopoeias throughout Europe (e.g., see Kujawska et al., 2017). This type of folk illness was however forgotten on other islands.

### Changes

The use of medicinal plants in the Adriatic islands has undergone dramatic changes due to shifts in lifestyle. Previous economic activities oriented toward fishery and agriculture (mainly viticulture, olive growing and sheep husbandry) has given way to a lifestyle dominated by dependence on tourism. The population is aging and composed mainly of retired people; many return to the islands from a life-long stay in Zagreb, Split, Rijeka, Zadar, the United States or Australia. Many of them are sailors-skippers of private yachts in France or Monaco. An important factor in changes in the local plant use, apart from the smaller connection to nature, is the disappearance of habitats suitable for collecting herbs. Most medicinal herbs in the islands, such as *Salvia officinalis, Hypericum perforatum, Ruta graveolens, Satureja montana* etc. have been associated with open rocky habitats grazed by sheep. Nowadays, maquis and *Pinus halepensis* encroach on the former pastures and even the most common aromatic herbs become rare. Ruderal weed habitats are also becoming species-poor due to the use of herbicides in orchards and the cessation of cultivation of wheat. *Matricaria chamomilla*, which was once collected from semi-spontaneous localities in gardens, now has to be sown every year. The lack of wild herbs has shifted the use of medicinal plants to those that come from the garden, where Lamiaceae plants dominate. Some new uses reflect general fashions in the whole area of south-eastern Europe, e.g., the popularity of *Helichrysum italicum* oil makes the species more popular as a part of herbal and liqueur mixes. The same goes for olive leaf herbal tea.

The most commonly used medicinal plants are used throughout the area. However, some regionalisms can be observed. For example *Parietaria judaica, Elymus repens, Plantago* spp. and *Ruta graveolens* are used mainly in the northern part of the study area. It must be stressed that the large majority of plants used in the islands, apart from some alien species, have been present in the European medical tradition since the times of Dioscorides (*see* e.g., Dioscorides et al., 2000; Leonti and Verpoorte, 2017; Krnjak, 2019).

### Comparison With Other Areas

The list of medicinal taxa used – 131 species - is not very long, taking into consideration the fact that it is spread over a few hundred kilometers and divided into islands with different histories. For comparison, Papageorgiou et al. (2020) recorded the harvesting of 144 species of medicinal plants just on one island (Lemnos) in Greece, and Axiotis et al. (2018) found 109 species of medicinal importance on only nine east Egean islands. Vitasović-Kosić et al. (2017) found 90 species used in Ćićarija, a small part of Istria in mainland Croatia, and Varga et al. (2019) found 83 species of medicinal plants used in a small region of inland Dalmatia. The unifying factor for the pharmacopoeias must be the fact that the islands have a very similar climate and vegetation, most of them composed of typical Mediterranean maquis, agricultural land and remnants of *Quercus ilex* forests. Only the westernmost Kvarner island has a slightly cooler climate with submediterranean influences.

### Insularity

In order to establish the extent to which local pharmacopoeias are impoverished by the insular character of the region, we tried to create a model for island pharmacopoeia that would take into account all the phenomena associated with being on an island that we observed or heard about from our informants (Table 6). We divided these forces into three domains. The first one is all the phenomena that might increase the richness of uses of plants due to their island location. Here we can mention limited access to biomedical services, which can help to maintain the tradition of local herbal medicine. Another is the possibility of long distance influence from sailors who bring new plants and uses from far-away countries. For example, the use of *Aloysia citriodora* is very widespread in Korčula. The plant is little known in the other islands. Specialized use can also stem from the existence of endemic plants (Redžić, 2009). The only endemic plant we found in use is *Satureja visianii* (Nikolić et al., 2020). The use of endemic plants is definitely more possible in archipelagos which were isolated from the mainland longer than the Croatian islands, e.g., in the islands of Greece (e.g., Axiotis et al., 2018). The majority of plants used in our study are common wild and cultivated pan-Mediterranean taxa. Isolation from the mainland also helps to give a local character to plant uses, especially given that endogamy in human populations is very strong on the islands. This is in contrast to the mainland of southern Croatia, where exogamy is very common – men have found wives from different villages – but as we found in our previous studies (Dolina et al., 2016), it is always the woman who moves to the man’s village (virilocality). This helps to exchange information on plants between villages – it is transferred between daughters-in-law and mothers-in-law from different villages. On the other hand endogamy, prevalent on the islands, can also conserve unique plant uses without bringing any new influence.

**TABLE 6:** Hypothetical driving forces of island pharmacopoeias for further testing.

Decreased BIOCULTURAL diversity	Neutral DIFERENTIATING FORCES (they may differentiate uses from other areas but not necessarily increase or decrease the number of species used	Increased BIOCULTURAL diversity
Local plant populations are small, therefore extremely sensitive to extinction due to overharvesting	Human endogamy in island populations	Preserving relic uses in most remote areas
Local plant populations extremely sensitive to extinction due to small population sizes and isolation	Floristic endemism	Possibility of importing uses and useful plants from distant countries via sea journeys
Influence of tourism as an easy source of income that distracts from traditional livelihoods causing loss of interest in the use of local plants	Geographical isolation	Influence of tourism and migration of retired people who lived outside the islands as a source of inspiration
Concentration on sea resources rather than agriculture and pastoralism causing loss of interest in the use of local plants		Possible absence of local ethnomedicinal specialists influenced by literature knowledge
Stochastic loss of knowledge about some plants due to small human population		Lower access to biomedicine compared to the mainland

The different driving forces of diversity presented, some more advantageous for larger islands, some for the small, are probably responsible for the fact that overall correlations between the studied variables were not significant (Table 5). It was rather the historical and social trajectories of each island that are more important. The similarity of neighboring islands with each other (*see* Figure 4) suggests that there has been an exchange of medicinal knowledge between them. This comes as no surprise, as very often two fishing villages from two islands facing each other were often involved in common fishing enterprises, which resulted in an exchange of marriage partners. We recorded such stories for Hvar and Vis, Pag and Rab etc.

One of the most important forces influencing local pharmacopoeas on some islands is the presence of local herbal specialists. The presence of such individuals may help maintain the general knowledge of medicinal herbs, but may also have a homogenizing effect if their knowledge is influenced by popular literature. On a few middle-sized islands, we have found influential local amateur herbalists who serve as the main hub of knowledge circulation on a given island. It is them who experiment with new species (often based on data from literature) or teach people how to name plants. For example, Mr Ivan Bamba Čumbelić on Mljet is active not only in this domain but is generally a social institution for this island. On Lastovo, Mr Giovanni Santi, who originates from the island, returned there after living abroad, retired and started an essential oil distillery as well as building a healing pyramid. On Šolta, Mr Dinko Sule publishes a local periodical devoted to the island’s history, culture and biology (*Bašćina*). He is also both a reservoir of past uses and an influential person on the island. Practically every conversation on plants with the locals started with them directing us to their local herb specialist. However, a large proportion of the islands, particularly the small ones, are devoid of individuals who are particularly interested in medical herbs, and people now only use or know a few basic plants. Similarly to the domain of wild vegetables (Łuczaj et al., 2019a), Korčula stands out in the medicinal plant domain as an island with a very vivid local community interested in preserving their tradition and using local resources. This island not only has the most widespread use of wild vegetables and the longest list of wild vegetables used but also the longest list of medicinal herbs in active use. Interestingly, also a small island of Šipan in the same sub-archipelago showed a very high level of knowledge of its inhabitants expressed by a large mean number of species listed.

### Limitations

The limitation of our study was an unequal number of interviews made in the islands. Nowadays, only a portion of the population claim to use medicinal plants, hence the difficulties in providing a larger number of interviews. We did not want to artificially set the number of interviews the same and rather chose to go for a saturation point when finding a new knowledgeable informant proved difficult. We believe that in many smaller islands we interviewed all such individuals.

## Data Availability

The datasets presented in this study can be found in online repositories. The name of the repository can be found below: http://repozytorium.ur.edu.pl/handle/item/5756. Voucher specimen numbers are in Table 2.
